# Tragic End of John Hunter

**Published:** 1857-02

**Authors:** 


*Tragic End of John Hunter.*—This distinguished surgeon, as our readers are aware, was attached to St. George's Hospital, London, and his temper was often sorely tried by his colleagues as well as by the governors of the hospital, who bitterly opposed what he termed his "innovations." Hunter had long been a sufferer from cardiac disease, and dreaded the effects of mental excitement, to which his irritable temperament rendered him constantly exposed. The last act of his enemies, and its melancholy result, are thus recorded by his biographer, Mr. Ottley:

"A committee was subsequently appointed to draw up a code of rules for regulating the admission and instruction of pupils; and a set of proposals was submitted to them by Mr. Hunter's colleagues, which was agreed to without his having been even consulted on the occasion! Many of the regulations adopted continue in force to this day; others, and especially those relating to the better instruction of the pupils, soon fell into disuse; and some seem to have been especially directed against Mr. Hunter. Amongst these latter, was one which determined that for the future, no person should be admitted as a student of the hospital without bringing certificates that he had been educated to the profession; a regulation which was probably designed to exclude Mr. Hunter's countrymen, who sometimes came up to town recommended to him, and entered as his pupils at the hospital, without having had any previous medical instruction. Nor was this clause long in taking effect; for in the autumn two young men, who had come up to town ignorant of this new regulation, applied to Hunter to be admitted under him at the hospital. He informed them of the law which had been passed, but undertook to press for their admission at the next Board-day, and directed them to furnish him with a statement of their case in writing. On the 16th of October the Board was to meet, and Hunter proposed to fill his promise, though he was so well aware of the risk he incurred by undertaking a task which he felt would agitate him, that in mentioning the circumstance to a friend who called on him in the morning, he expressed his apprehension lest some unpleasant dispute might occur, and his conviction that if it did, it would certainly prove fatal to him. At his accustomed hour he left his house to commence his morning rounds, and by accident forgot to take with him his list of appointments; he had left the house but a few moments when it was discovered, and Mr. Clift, who was then residing in his house, hastened with it to York Street, St. James, the first place on the list, where he found the carriage waiting. Hunter soon made his appearance, took the list, and in an animated tone called to the coachman to drive to St. George's. Arrived at the hospital, he found the Board already assembled, and entering the room, presented the memorial of the young men, and proceeded to urge the propriety of their being admitted. In the course of his remarks, he made some observations which one of his colleagues thought it necessary instantly and flatly to contradict. Hunter immediately ceased speaking, retired from the table, and struggling to suppress the tumult of his passion, hurried into an adjoining room, which he had scarcely reached, when, with a deep groan, he fell lifeless into the arms of Dr. Robertson, one of the physicians of the hospital, who chanced to be present. Dr. Baillie had immediately followed him from the Board-room, and Mr. Hume, who was in the house, was also summoned to his assistance. Various attempts were made for upwards of an hour to restore animation, under the hope that the attack might prove to be a fainting fit, such as he had before experienced, but in vain; life had fled; and all their efforts proving useless, his body was placed in a sedan chair and conveyed to Leicester-square, followed by his now vacant carriage."—*Western Lancet.*

